# Case mix, outcome and activity for patients admitted to intensive care units requiring chronic renal dialysis: a secondary analysis of the ICNARC Case Mix Programme Database

**DOI:** 10.1186/cc5785

**Published:** 2007-04-23

**Authors:** Colin A Hutchison, Alex V Crowe, Paul E Stevens, David A Harrison, Graham W Lipkin

**Affiliations:** 1 University Hospital Birmingham NHS Foundation Trust, Queen Elizabeth Medical Centre, Edgbaston, Birmingham, B15 2TH, UK; 2 Countess of Chester Hospital, Countess of Chester Health Park, Liverpool Road, Chester, Cheshire CH2 1UL, UK; 3 Department of Renal Medicine, Kent and Canterbury Hospital, Ethelbert Road, Canterbury, Kent CT1 3NG, UK; 4 Intensive Care National Audit & Research Centre (ICNARC), Tavistock House, Tavistock Square, London WC1H 9HR, UK

## Abstract

**Introduction:**

This report describes the case mix, outcome and activity for admissions to intensive care units (ICUs) of patients who require prior chronic renal dialysis for end-stage renal failure (ESRF), and investigates the effect of case mix factors on outcome.

**Methods:**

This was a secondary analysis of a high-quality clinical database, namely the Intensive Care National Audit & Research Centre (ICNARC) Case Mix Programme Database, which includes 276,731 admissions to 170 adult ICUs across England, Wales and Northern Ireland from 1995 to 2004.

**Results:**

During the eight year study period, 1.3% (*n *= 3,420) of all patients admitted to ICU were receiving chronic renal dialysis before ICU admission. This represents an estimated ICU utilization of six admissions (32 bed-days) per 100 dialysis patient-years. The ESRF group was younger (mean age 57.3 years versus 59.5 years) and more likely to be male (60.2% versus 57.9%) than those without ESRF. Acute Physiology and Chronic Health Evaluation II score and Acute Physiology Score revealed greater severity of illness on admission in patients with ESRF (mean 24.7 versus 16.6 and 17.2 versus 12.6, respectively). Length of stay in ICU was comparable between groups (median 1.9 days versus 1.8 days) and ICU mortality was only slightly elevated in the ESRF group (26.3% versus 20.8%). However, the ESRF group had protracted overall hospital stay (median 25 days versus 17 days), and increased hospital mortality (45.3% versus 31.2%) and ICU readmission (9.0% vs. 4.7%). Multiple logistic regression analysis adjusted for case mix identified the increased hospital mortality to be associated with increasing age, emergency surgery and nonsurgical cases, cardiopulmonary resuscitation before ICU admission and extremes of physiological norms. The adjusted odds ratio for ultimate hospital mortality associated with chronic renal dialysis was 1.24 (95% confidence interval 1.13 to 1.37).

**Conclusion:**

Patients with ESRF admitted to UK ICUs are more likely to be male and younger, with a medical cause of admission, and to have greater severity of illness than the non-ESRF population. Outcomes on the ICU were comparable between the two groups, but those patients with ESRF had greater readmission rates, prolonged post-ICU hospital stay and increased post-ICU hospital mortality. This study is by far the largest comparative outcome analysis to date in patients with ESRF admitted to the ICU. It may help to inform clinical decision-making and resource requirements for this patient population.

## Introduction

End-stage renal failure (ESRF) is a common, chronic disorder. Advances in dialysis services over recent years have resulted in patients living increasingly independent and healthier lives. Despite this, patients with ESRF are prone to repeated hospital admissions, some of which require admission to an intensive care unit (ICU). These admissions are predominantly related to the comorbidities associated with ESRF; of these, vascular access related infection and cardiovascular disease are the most common causes of admission to hospital [1].

A number of factors have led to a rapidly expanding ESRF population. Chief among these are issues such as increased life expectancy, resulting in the average age of the population rising, and the expanding population with predisposing chronic diseases such as diabetes mellitus [2,3]. The UK Renal Registry estimates the current incidence and prevalence of dialysis-dependent ESRF to be around 100 and 700 per million of the UK population, respectively.

Although it is perceived that the need for critical care services in the ESRF population is high and it is expected that this need will continue to increase [4], there is no adequate estimate of the actual critical care services needed. Moreover, there is no planning for critical care resource requirement to service the current ESRF population. Until recently, it was assumed that patients with ESRF admitted to critical care have considerably increased morbidity and mortality in comparison with the general ICU admission population. The recognized high ICU mortality of patients who develop acute renal failure (ARF) may in some cases be influencing the decision to admit to the ICU patients who require dialysis for ESRF. This assumption could lead to therapeutic nihilism limiting access to critical care for the ESRF population. Recently, studies including limited numbers of patients have examined this issue. Two [5,6] suggested that in fact the mortality of the ESRF population in the critical care setting is only moderately raised above the non-ESRF patient group, and nothing like the increased mortality seen with ARF. A third report, however, suggests that patients with ESRF in the critical care setting do have significantly increased mortality [7]. These reports also raise concerns about the predictive value of general ICU severity scoring systems to predict outcome in patients with ESRF in the critical care setting [5,6].

The need for high-quality data on outcomes, and the factors that are predictive of them, in ESRF patients in the critical care setting is required to confirm or refute these previous findings. Availability of such data will help to inform service planning and guide clinical decision making in this patient population. In the present study a large, high-quality, clinical database was used to identify admissions to ICUs across England, Wales and Northern Ireland of patients with ESRF who were already receiving chronic dialysis. We report, for the first time, national, baseline information that will be useful for both local benchmarking and for dictating future policy. This report describes case mix and factors that are predictive of outcome in patients with ESRF admitted to the ICU, as a first step toward achieving the desired service goals.

## Materials and methods

### Case Mix Programme Database

The Case Mix Programme (CMP) is a national comparative audit of adult, general critical care units in England, Wales and Northern Ireland coordinated by the Intensive Care National Audit & Research Centre (ICNARC). Data were extracted for 276,731 admissions to 170 intensive care units (ICUs) from the CMP Database, covering the period from December 1995 to January 2004. Details of the data collection and validation were reported previously [8].

### Selection of cases

Admissions were identified by the recording of the need for chronic renal replacement therapy, as part of the chronic health conditions for Acute Physiology and Chronic Health Evaluation (APACHE) II scoring [9]. The need for chronic renal replacement therapy is defined as, 'admission currently requires chronic renal replacement therapy (either chronic haemodialysis, chronic haemofiltration, or chronic peritoneal dialysis) for irreversible renal disease', and must be documented before admission or on admission to the CMP unit.

### Data

Data were extracted on case mix, outcome and activity, as defined below.

#### Case mix

Age at admission and sex were extracted. Admissions of patients who were mechanically ventilated during the first 24 hours in the ICU were identified by recording of mechanical ventilation on admission to the unit or by recording of a lowest or highest ventilated respiratory rate during the first 24 hours after admission. The following physiological variables, selected *a priori*, were extracted from records of the first 24 hours in the ICU: highest serum creatinine, lowest serum albumin and lowest haematocrit.

Acute severity was measured using the APACHE II Acute Physiology Score and the APACHE II score [9]. The former encompasses a weighting for acute physiology (defined by derangement from the normal range for 12 physiological variables during the first 24 hours in the ICU). The latter additionally encompasses a weighting for age and for past medical history of specified serious conditions.

Surgical status was defined as either nonsurgical, elective surgery, or emergency surgery, based on the source of admission to the CMP unit and the National Confidential Enquiry into Perioperative Deaths (NCEPOD) classification of surgery, as was previously described [8].

Organ system failures were assessed according to the method proposed by Knaus and coworkers [10], based on physiological data from the first 24 hours in the ICU. The organ system failures assessed are cardiovascular failure, respiratory failure, renal failure, haematological failure and neurological failure. Note that all patients on chronic renal dialysis are excluded from the renal failure category, and so admissions in the study population had a possible range from zero to four organ system failures.

#### Outcome

Survival data were extracted at discharge from the CMP unit and at ultimate discharge from hospital.

#### Activity

Length of stay in ICU was calculated in fractions of days from the dates and times of admission and discharge from the CMP unit. Length of stay in hospital was calculated in days from the dates of original admission to and ultimate discharge from an acute hospital. Transfers in from another ICU were identified as admissions whose source of admission to the CMP unit was ICU in the same or other hospital. Readmissions to ICU within the same hospital stay were identified from the postcode, date of birth and sex, and confirmed by the participating units. Treatment withdrawal was defined as the documented decision to withdraw all active treatment, other than comfort measures. The destination following discharge from the CMP unit was also extracted for all admissions of patients who were discharged alive.

### Analyses

Case mix, outcome and activity were described for all patients admitted who required chronic renal dialysis and for the remainder of the CMP Database, excluding admissions of patients for whom there was no evidence available to assess past medical history. The primary reason for admission to the CMP unit (coded using the ICNARC Coding Method [11]) was tabulated for patients requiring chronic renal dialysis. Ultimate hospital mortality, by number of organ system failures, was compared for patients requiring and not requiring chronic renal dialysis.

The outcomes of patient admitted who required chronic renal dialysis, as compared with other patients, adjusted for case mix factors, were assessed with a multiple logistic regression model on ultimate hospital mortality. Case mix adjustment was performed including the following factors: age, sex, surgical status, APACHE II chronic health conditions (excluding chronic renal replacement therapy), cardiopulmonary resuscitation (CPR) during 24 hours before admission to the CMP unit, Glasgow Coma Score (lowest during the first 24 hours in the CMP unit or the pre-sedation value for patients who were sedated or paralyzed and sedated for the first 24 hours), number of organ system failures, sepsis (defined physiologically using data from the first 24 hours following admission to the CMP unit [12]) and all of the physiological variables included in the APACHE II model plus serum albumin. Age, Glasgow Coma Score and number of organ system failures were modelled as having a linear effect on the log odds. All other variables were modelled categorically, using the categories from APACHE II or APACHE III [13] as appropriate for the physiological variables, but fitting new weights to each category. When a variable was present in both APACHE II and APACHE III, the categorization giving the greatest number of categories was selected. Categories from APACHE II were used to model temperature, mean arterial pressure, arterial pH, serum sodium, serum potassium, serum creatinine, haematocrit and white blood cell count. Categories from APACHE III were used to model heart rate, respiratory rate, oxygenation (either arterial to alveolar oxygen difference or arterial oxygen tension, depending on the fractional inspired oxygen level) and serum albumin. Patients whose records were lacking age, sex, surgical status, or any routinely measured physiological variables (temperature, blood pressure, heart rate, or respiratory rate) were excluded from the modelling. All other missing values were assumed to be normal and were placed in the category corresponding to zero APACHE II/III points.

The same multiple logistic regression approach was used to model the effects of the above parameters on ultimate hospital mortality within the group of patients requiring chronic renal dialysis. Because this involved a much smaller number of admissions, the APACHE II/III categories were first collapsed by combining adjacent categories such that each category contained at least 50 admissions. Results of this model were compared with the same model fitted in the group of patients not requiring chronic renal dialysis by introducing interaction terms.

All logistic regression models were assessed for discrimination by the area under the receiver operating characteristic (ROC) curve [14], and for overall fit by Brier's score (mean square error between outcome and prediction) [15] and Shapiro's *R *statistic (geometric mean probability assigned to the event that occurred) [16].

The usefulness of the newly-developed ESRF-specific model in discriminating between survivors and nonsurvivors among ESRF patients and non-ESRF patients was assessed using ROC curves. The utility of the model was also compared with the performance of the APACHE II score in these groups.

All analyses were performed using Stata 8.2 (StataCorp LP, College Station, TX, USA).

## Results

### Data

**Figure 1:**
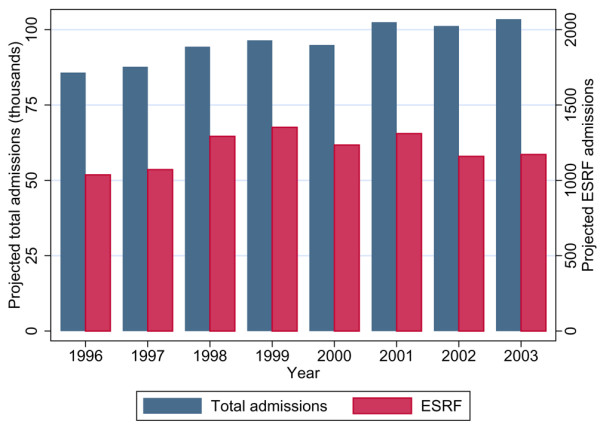
Projected total admissions to ICU and number requiring chronic renal dialysis. The figures relate to England, Wales and Northern Ireland. ESRF, end-stage renal failure (requiring chronic renal dialysis); ICU, intensive care unit.

Of 276,731 patients admitted to 170 adult ICUs in the CMP Database, for 270,972 (97.9%) there was sufficient evidence to assess past medical history. Of these, 3,420 (1.3%) were identified as requiring chronic renal dialysis. Figure 1 shows projected ICU admissions for the chronic renal dialysis population and the total population for the years of the study. In 2003, we project that there were 1,172 admissions to ICUs in England, Wales and Northern Ireland of patients requiring chronic renal dialysis, occupying a total of 5,920 ICU bed-days. The UK Renal Registry Report 2004 [17] estimated the total number of adult patients receiving renal replacement therapy in 2003 in England, Wales and Northern Ireland to be 33,929, of which 54% received dialysis. Based on these figures, ICU utilization in 2003 was six ICU admissions or 32 ICU bed-days per 100 dialysis patients. The ICU utilization by patients with ESRF remained stable over the past five study years, whereas the numbers of patients treated nationally for ESRF increased.

### Case mix, outcome and activity

Table 1 describes measures of case mix, outcome and activity for patients requiring chronic renal dialysis and admissions of all other patients for whom evidence was available to allow assessment of past medical history.

**Table 1:** Case mix, outcome and activity for patients admitted to ICUs requiring chronic renal dialysis as compared with other patients

	Parameter	Patients requiring chronic renal dialysis (*n *= 3,420)		Patients not requiring chronic renal dialysis (*n *= 267,552)		*P *value
Case mix	Age (mean [SD]; years)	57.3	(15.8)	59.5	(19.6)	< 0.001
	Male sex (*n *[%])	2,058	(60.2)	154,780	(57.9)	0.006
	CPR before admission (*n *[%])	466	(13.6)	19,535	(7.3)	< 0.001
	Mechanically ventilated (*n *[%])	2,107	(61.8)	167,840	(63.1)	0.135
	Highest serum creatinine (mean [SD]; mg per 100 ml/mmol per l])	6.5	(3.2)/575 (283)	1.5	(1.3)/133 (115)	< 0.001
	Lowest serum albumin (mean [SD]; g/l)	22.9	(7.7)	23.4	(8.5)	0.001
	Lowest haematocrit (%)/haemoglobin (g/dl) (mean [SD])	26.9	(5.9)/9.0 (2.0)	31.3	(6.6)/10.4 (2.2)	< 0.001
	APACHE II APS score^a ^(mean [SD])	17.2	(6.5)	12.6	(6.7)	<0.001
	APACHE II score^a ^(mean [SD])	24.7	(7.0)	16.6	(7.3)	< 0.001
	Surgical status (*n *[%])					< 0.001
	Nonsurgical	2,282	(66.7)	150,350	(56.2)	
	Elective surgery	592	(17.3)	66,017	(24.7)	
	Emergency surgery	545	(16.0)	50,947	(19.1)	
	Number of nonrenal organ system failures^b ^(*n *[%])					< 0.001
	None	1,156	(33.8)	107,140	(40.0)	
	1	1,223	(35.8)	99,299	(37.1)	
	2	743	(21.7)	46,447	(17.4)	
	3+	298	(8.7)	14,666	(5.5)	
Outcome	Mortality in ICU (*n *[%])	898	(26.3)	55,547	(20.8)	< 0.001
	Ultimate hospital mortality (*n *[%])	1,379	(45.3)	77,869	(31.2)	< 0.001
Activity	ICU LOS (median [IQR]; days)					
	Survivors	1.9	(0.9–4.2)	1.8	(0.9–4.5)	0.507
	Nonsurvivors	2.0	(0.6–6.0)	1.9	(0.7–6.1)	0.843
	Total hospital LOS (median [IQR]; days)					
	Survivors	25	(13–49)	17	(9–33)	< 0.001
	Nonsurvivors	15.5	(5–35)	8	(2–21)	< 0.001
	Transfers from another ICU (*n *[%])	120	(3.5)	10,508	(3.9)	0.210
	Readmissions within hospital stay (*n *[%])	306	(9.0)	12,676	(4.7)	< 0.001
	Treatment withdrawn (*n *[%])	364	(10.7)	26,119	(9.8)	0.087
	Destination following discharge (*n *[%])					< 0.001
	Ward, same hospital	1,788	(70.9)	155,487	(73.4)	
	Recovery, same hospital	14	(0.6)	975	(0.5)	
	ICU, same hospital	9	(0.4)	1,580	(0.8)	
	HDU, same hospital	295	(11.7)	28,695	(13.6)	
	Other intermediate care, same hospital	156	(6.2)	4,573	(2.2)	
	ICU, other hospital	94	(3.7)	10,898	(5.2)	
	HDU, other hospital	16	(0.6)	850	(0.4)	
	Other hospital, not ICU/HDU	137	(5.4)	6,088	(2.9)	
	Normal residence	12	(0.5)	2,662	(1.3)	

^a^Acute Physiology and Chronic Health Evaluation (APACHE) II exclusions: age < 16 years; intensive care unit (ICU) stay < 8 hours; readmissions within same hospital stay; transfers from another ICU; admissions following coronary artery bypass grafting; and admissions for primary burns. ^b^Organ system failures assessed physiologically according to the method of Knaus and coworkers [10]. APS, Acute Physiology Score; CPR, cardiopulmonary resuscitation; HDU, high dependency unit; IQR, interquartile range; LOS, length of stay; SD, standard deviation.

Patients requiring chronic renal dialysis were slightly younger than other patients (mean age 57.3 years versus 59.5 years) and were slightly more likely to be male (60.2% versus 57.9%). They were more likely to have received CPR during the 24 hours before admission to the CMP unit (13.6% versus 7.3%). They had greater creatinine (mean 6.5 mg/l versus 1.5 mg/l) and lower haematocrit (mean 26.9% versus 31.3%). Overall acute severity of illness was worse, as indicated by higher Acute Physiology Score (mean 17.2 versus 12.6) and APACHE II score (mean 24.7 versus 16.6). Overall, 67% of all patients requiring chronic renal dialysis were nonsurgical, as compared with 56% of other patients. The pattern of organ system failures was similar for both groups.

Crude mortality in the CMP unit was 26.3% for patients requiring chronic renal dialysis, as compared with 20.8% for other patients. At ultimate hospital discharge, mortality in these patients was 45.3% as compared with 31.2% in the reference group.

**Figure 2:**
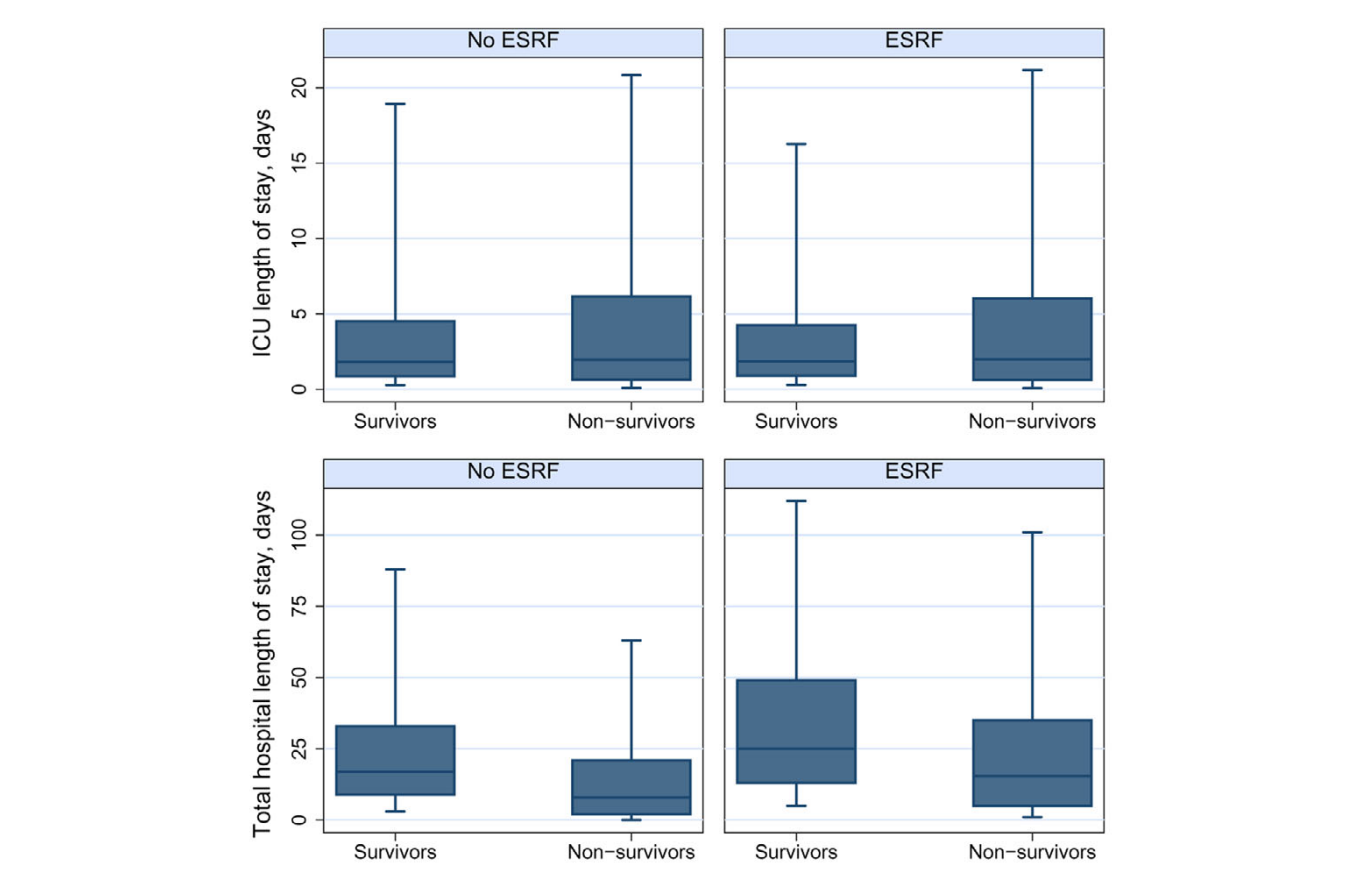
Length of stay in the ICU and in hospital. Box indicates median and quartiles; whiskers indicate 5th and 95th percentiles. ESRF, end-stage renal failure (requiring chronic renal dialysis); ICU, intensive care unit.

Patients requiring chronic renal dialysis had a similar length of stay in the CMP unit to that of other patients, but they had a longer stay in hospital (median 25 days versus 17 days for survivors; 15.5 days versus 8 days for nonsurvivors; Figure 2). Patients requiring chronic renal dialysis were more likely to be readmitted to the ICU during the same hospital stay (9.0% versus 4.7%), although the rate of direct transfers between ICUs was similar for the two groups of patients. There was no significant difference between the groups in the decision to withdraw treatment (9.8% versus 10.7% in non-ESRF and ESRF populations, respectively). The patterns of destination following discharge were broadly similar, although patients requiring chronic renal dialysis were slightly more likely to be transferred to high dependency care and were considerably more likely to be transferred to an 'other intermediate care area', which is the category containing renal units.

Of the 3,420 patients requiring chronic renal dialysis, 3,189 (93.2%) had a complete primary reason for admission specified, 230 (6.7%) had a partially coded reason for admission, and the remaining one admission (0.03%) had no reason for admission recorded. Of the 3,189 patients with a complete primary reason for admission, 275 (8.6%) had chronic renal failure recorded as the reason for admission (Table 2). The most common other reasons for admission were septic shock (179 [5.6%]) and pneumonia either with no organism isolated (167 [5.2%]) or a bacterial pathogen isolated (94 [2.9%]).

**Table 2:** Most common primary reasons for admission to the ICU for admissions requiring chronic renal dialysis

Primary reason for admission	*n *(%)	
Chronic renal failure	275	(8.6)
Septic shock	179	(5.6)
Pneumonia, no organism isolated	167	(5.2)
Bacterial pneumonia	94	(2.9)
Septicaemia	90	(2.8)
Status epilepticus or uncontrolled seizures	87	(2.7)
Cardiogenic pulmonary oedema	84	(2.6)
Hypovolaemic shock	84	(2.6)
Cardiogenic shock	79	(2.5)
CAPD related peritonitis	75	(2.4)

CAPD, Continuous ambulatory peritoneal dialysis; ICU, intensive care unit.

Hospital mortality increased steeply with number of organ system failures (Table 3). It was higher in patients requiring chronic renal dialysis, particularly at low numbers of organ system failures.

**Table 3:** Mortality by number of nonrenal organ system failures in patients requiring chronic renal dialysis as compared with other admissions

Number of nonrenal organ system failures^a^	Ultimate hospital mortality (deaths/admissions [%])	
	
	Admissions requiring chronic renal dialysis	Admissions not requiring chronic renal dialysis
0	289/1,036 (27.9)	14,825/100,125 (14.8)
1	469/1,077 (43.5)	28,071/92,242 (30.4)
2	413/662 (62.4)	24,118/43,545 (55.4)
3	172/228 (75.4)	9,967/12,907 (77.2)
4	36/44 (81.8)	888/997 (89.1)

^a^Organ system failures assessed physiologically, according to the method of Knaus and coworkers [10].

### Case mix adjusted effect of chronic renal dialysis on ultimate hospital mortality

After adjusting for case mix factors of age, sex, surgical status, APACHE II physiology variables, serum albumin and the number of nonrenal organ system failures (see Materials and methods, above), the odds ratio for ultimate hospital mortality associated with chronic renal dialysis was 1.24 (95% confidence interval [CI] 1.13 to 1.37) as compared with a crude odds ratio before case mix adjustment of 1.82 (95% CI 1.69 to 1.96). The case mix adjusted model had an area under the ROC curve of 0.857 (95% CI 0.855 to 0.858), a Brier's score (*B*) of 0.138 and a Shapiro's *R *of 0.653 when assessed for all admissions.

### Relationship of case mix factors with ultimate hospital mortality

Table 4 presents the results of the multiple logistic regression analysis of case mix factors on ultimate hospital mortality in the group of chronic renal dialysis patients. The following factors were associated with increased odds of hospital mortality: older age, emergency surgery and nonsurgical cases (as compared with elective surgery), presence of other chronic health conditions, CPR during the 24 hours before admission to the CMP unit, hospital stays of longer than one week before admission to the CMP unit, lower mean arterial pressure, high heart rate, high respiratory rate, extreme oxygenation values (high alveolar to arterial oxygen difference or low arterial oxygen tension), low arterial pH, low serum sodium, low serum albumin, extreme (high or low) white blood count, low Glasgow Coma Score, increasing number of organ system failures, and sepsis during the first 24 hours in the CMP unit. Among patients requiring chronic renal dialysis, this model had discrimination and fit statistics as follows: area under the ROC curve 0.817 (95% CI 0.802 to 0.832), *B *= 0.173 and *R *= 0.595.

**Table 4:** Effects of age, sex, surgical status, APACHE II physiological variables, serum albumin and number of organ system failures on ultimate hospital outcome in patients requiring chronic renal dialysis

Parameter	Patients requiring chronic renal dialysis					Admissions not requiring chronic renal dialysis	
	
	Deaths	*n*	(%)	Adjusted OR (95% CI)	*P *value^e^	Adjusted OR (95% CI)	*P *value^f^
Age (years)*				1.28 (1.20–1.36)	< 0.001	1.50 (1.49–1.52)	<0.001
< 45	223	681	(32.8)	per 10-year increase		per 10-year increase	
45–54	234	499	(46.9)				
55–64	314	711	(44.2)				
65–74	398	805	(49.4)				
75+	210	351	(59.8)				

Sex					0.145		0.653
Female	546	1,220	(44.8)	Reference		Reference	
Male	833	1,827	(45.6)	1.15 (0.95–1.38)		1.10 (1.07–1.12)	

Surgical status*					< 0.001		<0.001
Elective surgery	124	548	(22.6)	Reference		Reference	
Emergency surgery	194	486	(39.9)	1.69 (1.23–2.32)		2.49 (2.40–2.59)	
Nonsurgical	1,061	2,012	(52.7)	2.10 (1.59–3.78)		3.83 (3.69–3.97)	

Past medical history*					0.042		0.111
Absent	1,121	2,563	(43.7)	Reference		Reference	
Present	258	484	(53.3)	1.29 (1.01–1.64)		1.57 (1.52–1.62)	

CPR before admission*					< 0.001		0.424
No	1,083	2,621	(41.3)	Reference		Reference	
Yes	295	423	(69.7)	1.90 (1.44–2.52)		2.14 (2.05–2.22)	

LOS before admission (days)*					< 0.001		0.053
0	378	863	(43.8)	Reference		Reference	
1–2	198	595	(33.3)	0.76 (0.58–1.00)		0.99 (0.96–1.02)	
2–3	94	240	(39.2)	0.98 (0.69–1.40)		1.09 (1.04–1.14)	
3–6	197	448	(44.0)	0.95 (0.71–1.27)		1.29 (1.24–1.34)	
7+	511	900	(56.8)	1.95 (1.52–2.49)		1.86 (1.80–1.93)	

Temperature^a ^(°C)					0.783		0.078
< 34	80	126	(63.5)	1.24 (0.77–1.99)		1.96 (1.84–2.08)	
34–36	456	946	(48.2)	1.05 (0.85–1.30)		1.23 (1.19–1.26)	
36–38.5	465	1,208	(38.5)	Reference		Reference	
38.5–39	120	274	(43.8)	1.16 (0.85–1.59)		0.95 (0.91–0.98)	
≥ 39	193	382	(50.5)	1.13 (0.84–1.51)		1.10 (1.06–1.13)	

Mean arterial pressure^a ^(mmHg)*					< 0.001		<0.001
< 50	410	596	(68.8)	1.96 (1.36–2.83)		1.96 (1.87–2.04)	
50–70	575	1,189	(48.4)	1.29 (0.95–1.75)		1.21 (1.17–1.25)	
70–110	117	351	(33.3)	Reference		Reference	
110–130	144	496	(29.0)	0.74 (0.52–1.05)		0.96 (0.92–1.00)	
≥ 130	100	357	(28.0)	0.62 (0.42–0.92)		1.26 (1.20–1.32)	

Heart rate^b ^(beats/min)*					< 0.001		0.638
< 50	86	168	(51.2)	1.34 (0.88–2.06)		1.15 (1.08–1.22)	
50–100	242	780	(31.0)	Reference		Reference	
100–110	170	446	(38.1)	1.23 (0.92–1.64)		1.11 (1.07–1.16)	
110–120	228	486	(46.9)	1.57 (1.18–2.08)		1.37 (1.32–1.42)	
120–140	373	697	(53.5)	1.81 (1.40–2.36)		1.72 (1.66–1.77)	
140–155	137	241	(56.9)	1.86 (1.30–2.66)		2.15 (2.06–2.24)	
≥ 155	107	162	(66.0)	2.09 (1.35–3.23)		2.52 (2.40–2.64)	

Respiratory rate^b ^(breaths/min)*					< 0.001		0.082
< 6	63	121	(52.1)	0.99 (0.60–1.64)		1.22 (1.15–1.30)	
6–12	357	881	(40.5)	1.11 (0.84–1.46)		1.07 (1.04–1.11)	
12–14	230	496	(46.4)	1.38 (1.01–1.88)		1.23 (1.19–1.27)	
14–25	207	503	(41.2)	Reference		Reference	
25–35	269	627	(42.9)	1.13 (0.84–1.51)		0.99 (0.96–1.03)	
35–40	115	190	(60.5)	2.11 (1.40–3.18)		1.25 (1.19–1.32)	
≥ 40	96	148	(64.9)	2.32 (1.46–3.68)		1.49 (1.41–1.56)	

Oxygenation^b ^(mmHg)*					0.025		0.003
A-aDO_2 _(FiO_2 _≥ 0.5)							
< 250	92	182	(50.6)	Reference		Reference	
250–350	187	332	(56.3)	1.28 (0.96–1.72)		1.28 (1.24–1.32)	
350–500	109	200	(54.5)	0.73 (0.49–1.07)		1.56 (1.48–1.63)	
≥ 500	164	242	(67.8)	1.26 (0.87–1.84)		1.70 (1.63–1.77)	
PaO_2 _(FiO_2 _< 0.5)							
< 50	33	61	(54.1)	1.53 (0.80–2.93)		1.06 (0.97–1.16)	
50–70	130	295	(44.1)	1.04 (0.77–1.41)		0.95 (0.92–0.99)	
70–80	128	343	(37.3)	0.78 (0.58–1.04)		0.91 (0.87–0.94)	
≥ 80	352	846	(41.6)	Reference		Reference	

Arterial pH^a^*					< 0.001		0.985
< 7.15	156	190	(82.1)	2.65 (1.68–4.18)		2.95 (2.78–3.13)	
7.15–7.25	157	248	(63.3)	1.53 (1.08–2.16)		1.65 (1.59–1.72)	
7.25–7.33	248	544	(45.6)	1.16 (0.92–1.47)		1.20 (1.16–1.23)	
7.33–7.5	559	1,380	(40.5)	Reference		Reference	
≥ 7.5	79	153	(51.6)	1.40 (0.95–2.07)		1.40 (1.33–1.48)	

Serum sodium^a ^(mmol/l)*					0.035		0.079
< 130	174	331	(52.6)	1.44 (1.09–1.90)		1.43 (1.37–1.48)	
130–150	1,069	2,468	(43.3)	Reference		Reference	
≥ 150	41	64	(64.1)	1.12 (0.61–2.07)		2.26 (2.14–2.39)	

Serum potassium^a ^(mmol/l)					0.180		<0.001
< 3	68	125	(54.4)	1.15 (0.74–1.78)		1.09 (1.04–1.14)	
3–3.5	199	379	(52.5)	1.16 (0.89–1.52)		0.99 (0.96–1.02)	
3.5–5.5	658	1,482	(44.4)	Reference		Reference	
5.5–6	143	351	(40.7)	0.91 (0.68–1.21)		1.26 (1.19–1.32)	
6–7	145	370	(39.2)	0.74 (0.55–0.99)		1.39 (1.31–1.48)	
≥ 7	68	153	(44.4)	0.84 (0.55–1.28)		1.40 (1.25–1.56)	

Serum creatinine^a ^(mg/100 ml)					0.295		0.095
< 1.5	21	73	(28.8)	Reference		Reference	
1.5–2	28	67	(41.8)	0.55 (0.28–1.08)		1.05 (1.02–1.09)	
2–3.5	152	307	(49.5)	0.90 (0.58–1.39)		1.37 (1.32–1.42)	
≥ 3.5	1,048	2,365	(44.3)	0.80 (0.55–1.15)		1.25 (1.18–1.32)	

Serum albumin^b ^(g/l)*					< 0.001		0.060
< 20	453	823	(55.0)	1.50 (1.20–1.87)		1.37 (1.34–1.41)	
20–25	239	501	(47.7)	1.35 (1.05–1.72)		1.00 (0.97–1.03)	
≥ 25	350	990	(35.4)	Reference		Reference	

Haematocrit^a ^(%)					0.133		0.002
< 20	164	324	(50.6)	0.70 (0.49–1.01)		1.26 (1.18–1.34)	
20–30	790	1,769	(44.7)	0.86 (0.70–1.06)		1.16 (1.14–1.19)	
≥ 30	326	751	(43.4)	Reference		Reference	

White blood count^a ^(× 1,000/mm^3^)*					0.021		0.884
< 3	92	129	(71.3)	2.02 (1.26–3.25)		1.70 (1.60–1.79)	
3–15	586	1,577	(37.2)	Reference		Reference	
15–20	252	533	(47.3)	1.05 (0.83–1.33)		1.13 (1.10–1.17)	
20–40	287	508	(56.5)	1.28 (1.00–1.64)		1.26 (1.22–1.30)	
≥ 40	32	48	(66.7)	1.34 (0.67–2.70)		1.38 (1.25–1.53)	

Glasgow Coma Score^c^*				1.12 (1.09–1.15)	< 0.001	1.09 (1.09–1.09)	0.067
3–6	360	466	(77.3)	per decrease of 1		per decrease of 1	
7–9	73	138	(52.9)				
10–12	77	161	(47.8)				
13–15	756	2,044	(37.0)				

Organ system failures^d^*				1.25 (1.07–1.46)	0.005	1.31 (1.28–1.33)	0.561
0	289	1,036	(27.9)	per increase of 1		per increase of 1	
1	469	1,077	(43.6)				
2	413	662	(62.4)				
3+	208	272	(76.5)				

Sepsis*					0.022		0.060
No	785	2,035	(38.6)	Reference		Reference	
Yes	594	1,012	(58.7)	1.28 (1.04–1.57)		1.04 (1.02–1.07)	

A total of 2,922 patients are included in this analysis. Significant factors (*P *< 0.05) in the model for admissions of patients requiring chronic renal dialysis are highlighted by asterisks. Exclusions are as follows: readmissions within the same hospital stay; and admissions missing age, sex, surgical status, temperature, mean arterial pressure, heart rate or respiratory rate. Those admissions with missing values for any other physiological variables were assumed to be normal and placed in the reference category. ^a^Categories from Acute Physiology and Chronic Health Evaluation (APACHE) II (reference category equivalent to zero APACHE II points). ^b^Categories from APACHE III (reference category equivalent to zero APACHE III points). ^c^Pre-sedation values used for admissions sedated or paralyzed during the first 24 hours in intensive care unit (ICU). ^d^Organ system failures assessed physiologically, in accordance with the method of Knaus and coworkers [10]. ^e^*P *value for significance of factor in the end-stage renal failure (ESRF) model. ^f^*P *value for difference between ESRF and non-ESRF models (test of interaction). A-aDO_2_, alveolar-arterial oxygen difference; CI, confidence interval; CPR, cardiopulmonary resuscitation; FiO_2_, fractional inspired oxygen; LOS, length of stay; OR, odds ratio; PaO_2_, arterial oxygen tension.

When compared with the same model fitted in patients not requiring chronic renal dialysis, a number of factors exhibited a significantly different relationship with hospital mortality. Factors with a weaker association with hospital mortality in the ESRF population were age, surgical status, oxygenation, potassium and haematocrit. Adjusting for all other factors, a high mean arterial pressure (≥ 130 mmHg) appeared to exhibit a protective effect in the ESRF population, whereas in the non-ESRF population it was harmful (odds ratio 0.62 versus 1.24).

### Discrimination of the APACHE II score and ESRF-specific model

**Figure 3:**
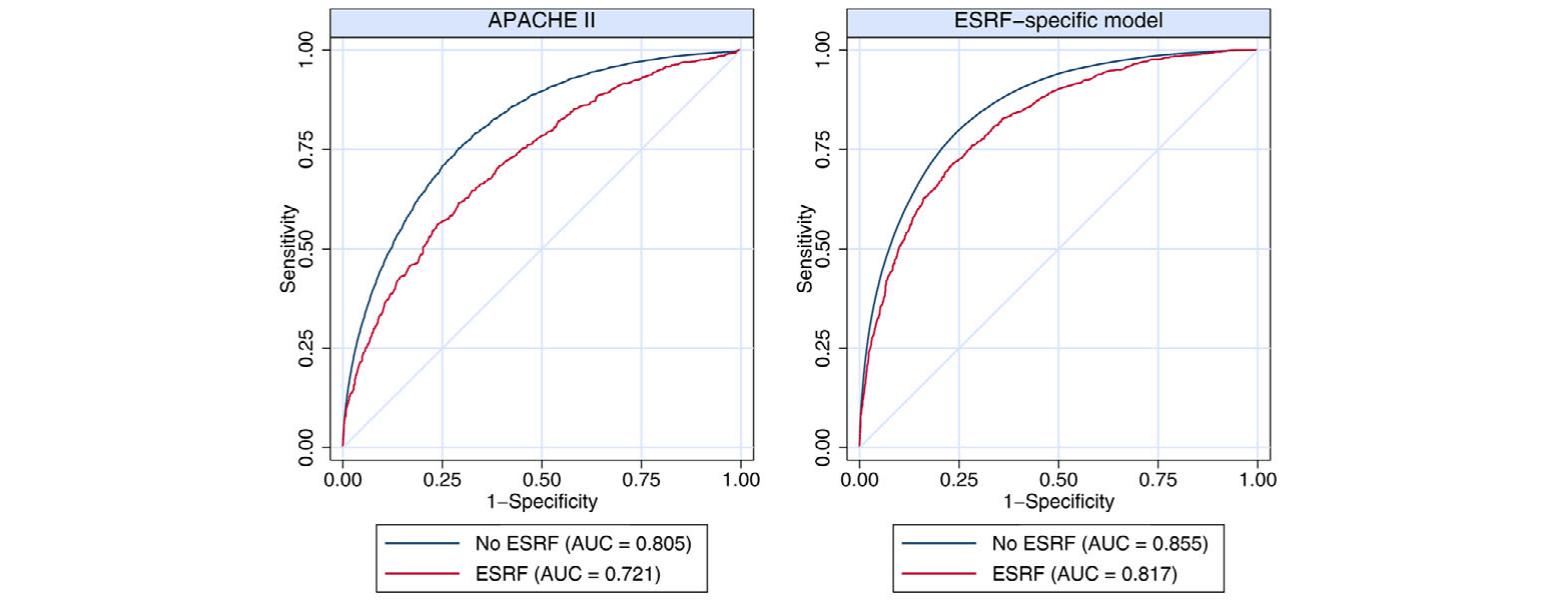
Comparison between APACHE II and the ESRF-specific model in discriminating between survivors and nonsurvivors. Shown are receiver operating characteristic curves for APACHE II and the ESRF-specific model from Table 4 for patients admitted to intensive care units requiring and not requiring chronic renal dialysis. APACHE, Acute Physiology and Chronic Health Evaluation; ESRF, end stage renal failure (requiring chronic renal dialysis).

The area under the ROC curve for the APACHE II score was 0.721 (95% CI 0.701 to 0.741) for the ESRF group as compared with 0.805 (95% CI 0.803 to 0.807) for the non-ESRF group (*P *< 0.001; Figure 3). This demonstrates that APACHE II scores are less sensitive in the ESRF population than in the non-ESRF population in discriminating between survivors and nonsurvivors. Discrimination was improved by using the new ESRF-specific model, but it was still worse among the ESRF group than in the non-ESRF group (area under the ROC curve 0.817 [95% CI 0.802 to 0.832] versus 0.853 [95% CI 0.851–0.854]; *P *< 0.001).

## Discussion

The aim of this study was to describe the case mix and outcome of adult admissions to ICU of patients with ESRF in the UK. To our knowledge four previous studies have reported on outcomes of patients with ESRF in the ICU, three of which were single centre and all of which included relatively small populations [5-7,18]. These highlighted the need for a large multicentre study to describe conclusively the admission of patients with ESRF to ICUs and their outcomes. Over the examined eight-year period, 1.2% (*n *= 3,420) of all patients admitted to the ICU (*n *= 276,731) had ESRF and were receiving dialysis (either chronic peritoneal or haemodialysis). This figure is considerably lower than the 3.7% observed in the USA [6] and 8.6% in a single centre study conducted in a French ICU [18]. This discrepancy is not surprising, given the limitations of single centre studies and the considerable differences in the utilization of both renal replacement therapy and ICU resources between different European and North American countries.

During the study period there was considerable expansion in the total number of admissions, but this was not matched by an expansion in the number of dialysis patients being admitted to ICU. This is particularly surprising because the total UK dialysis population increased by about 50% over the same time period and merits further investigation. Based on 2003 data, these figures give an annual ICU utilization of 1,172 admissions, or six admissions per 100 patients in the dialysis population. This compares to an overall ICU utilization of two admissions per 1,000 of the general population of England, Wales and Northern Ireland. It must be stressed that this utilization represents the current usage but not the need for ICU care among patients with ESRF, which is almost certainly greater and will rise as the population grows.

As seen in the study conducted by Dara and coworkers [5], admission to ICU of patients with ESRF is more common in men than women, which is consistent with the male predominance in the dialysis population. We found the ESRF population to be significantly younger than the non-ESRF population (mean age 57.3 years versus 59.5 years), which is in contrast to the work of Clermont and coworkers [6], who did not find a significant difference in age between ESRF and non-ESRF patients. This finding raises the possibility that there could be a denial of access to the ICU for the dialysis population on the basis of age. The greater serum creatinine and lower haematocrit observed in the dialysis population was not unexpected, possibly reflecting acute complications directly attributable to the underlying disease such as pulmonary oedema or hyperkalaemia.

In the present series, patients with ESRF were found to have greater severity of illness than the non-ESRF population on admission to the ICU, as defined by both the Acute Physiology Score (17.2 versus 12.6) and APACHE II score (24.7 versus 16.6); this is consistent with the findings of earlier studies [6,7,18]. This implies that ESRF patients are not being denied entry to ICU on the basis of severity of illness; rather, it raises the issue of whether late referral or acceptance of dialysis patients to ICU is influencing the findings. Some of this difference in severity of illness at admission between ESRF and non-ESRF patients could be explained by our findings that there was a significant difference in the disease aetiology between the two groups. There were significantly more nonsurgical admissions in the ESRF population (66.7% versus 56.2%), and a greater proportion of this group was admitted following CPR. It is not possible from our analysis to determine whether the ESRF group who required pre-admission CPR suffered a primary cardiac arrhythmia, the incidence of which is known to be increased in this patient population. The admission cause was coded as chronic renal failure in a small percentage of the ESRF cohort. This probably reflects a direct acute complication of renal failure such as volume overload or electrolyte disturbance.

Somewhat surprisingly, we found that the length of ICU stay was equivalent between the two groups of patients, despite the ESRF patients being sicker and having greater comorbidity. Many UK renal units have considerable experience in the care of critically ill patients with renal disease, thus allowing earlier ICU discharge. This finding is consistent with the findings of Clermont and coworkers [6]. However, the ESRF population had a significantly longer hospital stay following discharge from ICU (25 days versus 17 days in the non-ESRF group for hospital survivors). There was also a marked difference in readmission rates between the two groups (9.0% in ESRF patients versus 4.7% in non-ESRF patients). These figures could provide a basis for estimating the minimum service required to provide ICU services for the ESRF population and the costs of this service.

The large numbers included in the present study enabled us to achieve statistically significant confirmation of the suggestions made by Clermont [6] and Dara [5] and their coworkers, namely that the increased mortality observed in the ESRF population in the ICU setting is significantly lower than the increased mortality seen with ARF in the ICU setting. We found that the mortality at discharge from the ICU was 5.5% higher in the ESRF population (26.3% versus 20.8% in the non-ESRF population). This increased ICU mortality probably reflects the increased severity of illness seen in this population. However, the mortality rate was considerably lower than that seen in a contemporaneous population of patients for whom ARF was recorded as the primary cause of ICU admission in an analysis of the same database (43.3%) [19]. Although increased mortality is seen in the ESRF population admitted to ICU, the mortality rate is considerably lower than that seen in the ARF population.

The ultimate hospital mortality in the ESRF population was 45.3% (95% CI 43.5% to 47.0%), as compared with 31.2% (95% CI 31.0% to 31.4%) in the non-ESRF population. Although this represents a significantly increased mortality in the ESRF group, it once again is much lower than the ultimate hospital mortality seen in the population of patients with ARF as their primary reason for ICU admission in the same dataset (58.6%) [19]. This difference is even more marked when one examines subgroups of the ARF population, such as those patients presenting with oliguric ARF to the ICU, who have an ultimate hospital mortality of 70.3% [19]. When examining outcomes for number of organ system failures, we found the ESRF population to have a higher mortality with a lower number of nonrenal organ failures.

We demonstrated significant differences in outcome between the two groups in terms of increased hospital stay following discharge from the ICU, increased rate of readmission to ICU and increased ultimate hospital mortality. It is likely that there are multiple reasons for these differences in outcome between the two groups. These include issues such as lack of physiological reserve in the dialysis population, and the well described increased rates of hospital-acquired infections in the ESRF population, including a high rate of staphylococcal bacteraemia and *Clostridium difficile *infection [20-22]. There is also the ongoing need for vascular and peritoneal access, with their corresponding complications; all of these probably contribute to these differences in outcome. Drawing from these significant differences in outcome, it may be that current management of the ESRF population following ICU discharge is suboptimal in England, Wales and Northern Ireland. There may be a greater need for closer post-ICU monitoring and increased focus on intermediate care, including rehabilitation. This requires further investigation but it does raise the possibility that, with adequate resource planning and delivery, it may well be possible to reduce some of these differences.

Analysis of factors that affect outcome in the ESRF group revealed no real surprises. Significant factors were older age; surgical status; physiological extremes such as hypotension, bradycardia, tachypnoea and hypoxia; biochemical derangement with hyponatraemia, sepsis and leucopenia; and the number of additional nonrenal organ system failures. A number of the factors examined exhibited either a stronger or weaker relationship to outcome in the ESRF population than in the non-ESRF population.

Analysis of ROC curves demonstrated that the APACHE II score's discrimination of patient outcome in the ESRF population was worse than that among patients admitted to ICU who did not require dialysis. This is consistent with previous work in patients with ARF admitted to the ICU. Lins and coworkers [23] demonstrated that the APACHE II score is a less sensitive predictor of outcome in the ARF setting than are renal-specific scoring systems such as the Stuivenberg Hospital Acute Renal Failure system [23]. This should be considered in any clinical decision-making process in patients with ESRF being considered for admission to the ICU.

## Conclusion

Patients with dialysis-dependent ESRF who are admitted to UK ICUs are more likely to be younger and male, with a medical cause of admission, and to have greater severity of illness than the non-ESRF population. Despite this, ICU stay was similar and ICU mortality for patients with ESRF was only marginally increased. Nevertheless, patients with ESRF had increased ICU readmission rates, prolonged hospital stay and greater post-ICU mortality as compared with the general ICU population. Patient outcomes were considerably better than those reported for patients with ARF admitted to the ICU. This report may facilitate planning of adequate ICU resources for this population. It may also inform the clinical decision process surrounding ICU admission for patients receiving chronic dialysis therapy.

## Key messages

• ICU utilization by dialysis patients in the UK is approximately six admissions (32 bed-days) per 100 patients per year.

• Patients with ESRF admitted to ICU are more likely to be younger and male, with a medical cause for admission, and to have a greater severity of illness than are non-ESRF patients.

• Patients with ESRF have increased ICU readmission rates, prolonged hospital stay and increased post-ICU mortality (adjusted odds ratio 1.24 as compared with non-ESRF patients); however, mortality is considerably lower than for those patients with ARF who require ICU admission.

• The APACHE II score has lower sensitivity in discriminating between survivors and non-survivors in the ESRF population than in the non-ESRF population.

• These data allow, for the first time, estimation of minimum utilization of ICU services by the ESRF population in the UK and should facilitate service planning.

## Abbreviations

APACHE = Acute Physiology and Chronic Health Evaluation; ARF = acute renal failure; CMP = Case Mix Programme; CPR = cardiopulmonary resuscitation; ESRF = end-stage renal failure; ICNARC = Intensive Care National Audit & Research Centre; ICU = intensive care unit; OR = odds ratio; ROC = receiver operating characteristic.

## Competing interests

The authors declare that they have no competing interests.

## Authors' contributions

AVC, PES, DAH and GWL designed the study. DAH performed the analyses. CAH, DAH and GWL drafted the manuscript. All authors contributed to the interpretation of results and critical revision of the manuscript, and have read and approved the final manuscript.
